# Circulating miRNA-21 as a diagnostic biomarker in elderly patients with type 2 cardiorenal syndrome

**DOI:** 10.1038/s41598-020-61836-z

**Published:** 2020-03-17

**Authors:** Yan Wang, Yi Liang, WenJun Zhao, GuangPing Fu, QingQuan Li, XuChen Min, YiFang Guo

**Affiliations:** 1 Department of Geriatric Cardiology, Hebei General Hospital, Shijiazhuang, Hebei China; 2 Hebei Medical University, major in Cardiovascular Medicine, Shijiazhuang, Hebei China; 3 Hebei Key Laboratory of Forensic Medicine, Department of Forensic Medical, Hebei Medical University, Shijiazhuang, Hebei China; 4 Department of International Medical, the First Hospital of Shijiazhuang, Shijiazhuang, Hebei China

**Keywords:** Genetics, Diagnostic markers, Cardiovascular biology

## Abstract

Circulating miRNAs have attracted attention as serum biomarkers for several diseases. In this study, we aimed to evaluate the diagnostic value of circulating miRNA-21 (miR-21) as a novel biomarker for elderly patients with type 2 cardiorenal syndrome (CRS-2). A total of 157 elderly patients with chronic heart failure (CHF) were recruited for the study. According to an estimated glomerular filtration rate (eGFR) cut-off of 60 ml/min/1.73 m^2^, 84 patients (53.5%) and 73 patients (46.5%) were assigned to the CRS group and the CHF group, respectively. Expression levels of serum miR-21 and biomarkers for CRS, such as kidney injury factor-1 (KIM-1), neutrophil gelatinase-related apolipoprotein (NGAL), cystatin C (Cys C), amino-terminal pro-B-type natriuretic peptide (NT-proBNP), N-acetyl-κ-D-glucosaminidase (NAG), and heart-type fatty acid–binding protein (H-FABP), were detected. Serum miR-21, KIM-1, NGAL, Cys C, NT-proBNP and H-FABP levels were significantly higher in the CRS group than in the CHF group (*P* < 0.01), whereas NAG expression was not significantly different between the two groups (*P* > 0.05). Cys C, H-FABP and eGFR correlated significantly with miR-21 expression, but correlations with miR-21 were not significant for NT-proBNP, NGAL, NAG and KIM-1. Moreover, multivariate logistic regression found that serum miR-21, increased serum Cys C, serum KIM-1, hyperlipidaemia and ejection fraction (EF) were independent influencing factors for CRS (*P* < 0.05). The AUC of miR-21 based on the receiver operating characteristic (ROC) curve was 0.749, with a sensitivity of 55.95% and a specificity of 84.93%. Furthermore, combining miR-21 with Cys C enhanced the AUC to 0.902, with a sensitivity of 88.1% and a specificity of 83.6% (*P* < 0.001). Our findings suggest that circulating miR-21 has medium diagnostic value in CRS-2. The combined assessment of miR-21 and Cys C has good clinical value in elderly patients with CRS-2.

## Introduction

The term cardiorenal syndrome (CRS) refers to different clinical conditions in which heart and kidney dysfunction overlap. Ronco *et al*. proposed classifying CRS into 5 subtypes based on the potential underlying pathophysiologic mechanisms^1^. Among these subtypes, type 2 cardiorenal syndrome (CRS-2) is characterized by chronic abnormalities in cardiac function that result in kidney injury or dysfunction, along with clinical manifestations of CHF and an estimated glomerular filtration rate (eGFR) <60 ml/min/1.73 m^2^ ^2^. Furthermore, the incidence of renal insufficiency increases to 45–63% for elderly CHF patients with hypertension, diabetes, atherosclerotic heart disease and lipid metabolism diseases^3–5^. Assessment of kidney injury in CHF patients has previously been limited to serum creatinine, urea nitrogen and urinary protein excretion assays, which are prognostic for renal outcomes in CKD patients but not in heart failure patients^6^. However, novel kidney biomarkers (Cys C, NGAL, KIM-1, H-FABP and NAG) have recently been evaluated in CHF patients, and their serum levels have prognostic value for not only renal outcomes but also cardiovascular outcomes^7^. In addition, as an indicator of CHF, NT-proBNP correlates negatively with the eGFR level and can be used as an independent predictor of renal damage (i.e., eGFR <60 ml/min/1.73 m^2^)^8^.

MicroRNAs (miRNAs) are endogenous small noncoding RNAs of 21–24 nucleotides that control gene expression post-transcriptionally by inducing target miRNA destabilization or inhibiting protein translation. miRNAs play important roles in several diseases, such as heart failure, hypertension and kidney disease^9–12^, and many miRNAs have been identified as ideal candidate biomarkers due to their stable expression^12,13^. miR-21, which is encoded on chromosome 17q23.2, plays a regulatory role in several diseases, including cancer^14,15^, cardio-fibrosis^16,17^ and kidney disease^18,19^, and combining miR-21 with hs-TnT was demonstrated to significantly increase the diagnostic value in acute coronary syndrome (ACS) patients^20^. Several studies have reported that miR-21 suppresses mitochondrial biogenesis and amplifies kidney disease and fibrosis^21–23^. Moreover, molecular miRNA signalling pathways suggest that miR-21 is an excellent biomarker for certain treatments for the early stages of fibrosis and tissue remodelling^24–30^. Thus, circulating miR-21 has diagnostic and prognostic value for heart and renal diseases, which suggests that serum levels of circulating miR-21 may have value in the diagnosis of cardiorenal syndrome.

The aim of our study was to detect the level of serum circulating miR-21 in elderly patients with CRS-2 and to propose a better diagnostic and therapeutic biomarker for these patients by combining this level with new serum biomarkers.

## Results

### Patient demographics

As shown in Table 1, the mean age of the patients and the proportion of males were not significantly different between the CRS and CHF groups (all *P* > 0.05). The patients in the CRS group had a lower level of haemoglobin (HB) (*P* < 0.05), whereas no significant differences in white blood cell count (WBC), ejection fraction (EF) or the proportions of diabetes, hypertension, hyperlipidaemia, coronary atherosclerotic heart disease, myocardial infarction, and hypothyroidism were found between the two groups (all *P* > 0.05).

**Table 1:** Clinical characteristics of individuals CHF and CRS group.

Characteristics	CHF Group (n = 73)	CRS Group (n = 84)	*P*
Gender (male/female)	36/37	38/46	0.610
Age (years)	77 (12)	79 (10)	0.103
Hypertension	44	56	0.406
Diabetes Mellitus	21	25	0.891
Hypothyroidism	15	18	0.893
Hyperlipidemia	6	15	0.077
Coronary atherosclerotic heart disease	46	52	0.886
Myocardial Infarction	22	30	0.459
HB (g/L)	128 (21)	113.5 (27.75)	<0.001
WBC (1 × 10^9^)	6.27 (2.9)	6.29 (3.32)	0.553
EF (%)	55 (23)	46 (19.75)	0.086

Data are expressed as Median (Quartile range) WBC: white blood cell count, EF: ejection fraction, HB: haemoglobin.

### Expression of serum biomarkers between the CRS and CHF groups

**Figure 1:**
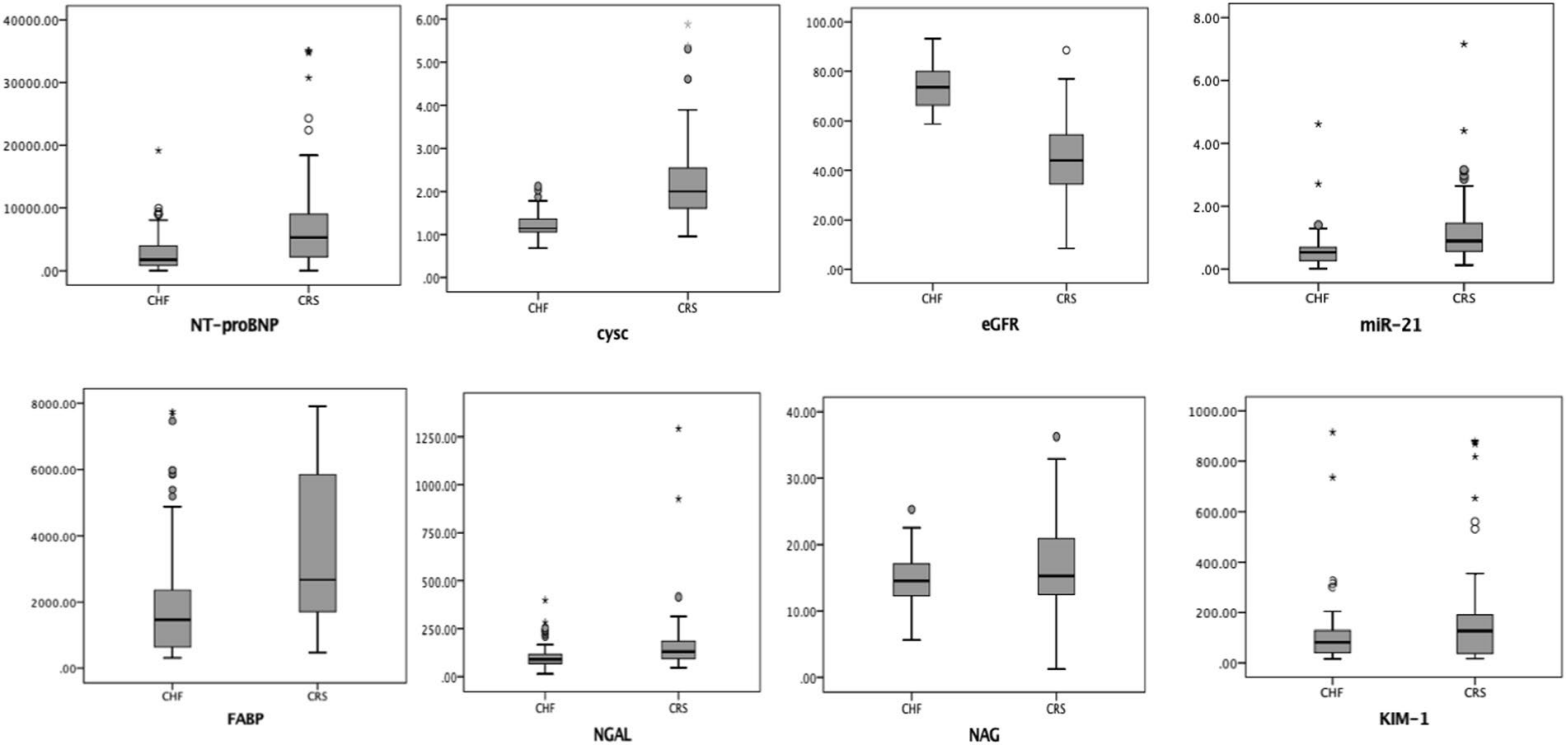
Expression of each biomarker in CHF and CRS serum. *CRS*: type 2 cardiorenal syndrome; *miR-21*: microRNA-21; *CHF*: chronic heart failure; *eGFR*: estimated glomerular filtration rate; *KIM-1*: kidney injury factor-1; *NGAL*: neutrophil gelatinase-related apolipoprotein; *Cys C*: cystatin C; *NT-proBNP*: amino-terminal pro-B-type natriuretic peptide; *NAG*: N-acetyl-κ-D-glucosaminidase; *H-FABP*: heart-type fatty acid-binding protein.

Compared with those in the CHF group, the expression levels of NT-proBNP [5332 (6820) vs 1739 (3332)], *P* < 0.001], KIM-1 [127.5 (157.76) vs 82.23 (94.05) *P* = 0.006], NGAL [130.15 (92.07) vs 89.3 (48.3), *P* < 0.001], Cys C [2.0 (0.95) vs 1.14 (0.31), *P* < 0.001], H-FABP [2677.9 (4191.8) vs 1469.97 (1739.88), *P* < 0.001] and miR-21 [0.89 (0.89) vs 0.53 (0.44), *P* < 0.001] were higher in the CRS group. Conversely, no significant differences between the two groups were found for NAG expression [15.28 (8.51) vs 14.5 (4.9), *P* = 0.166] (Table 2 and Fig. 1).

**Table 2:** Expression of the serum biomarkers between CRS and CHF groups.

Characteristics	CHF Group (n = 73)	CRS Group (n = 84)	*P*
NT-proBNP (ng/L)	1739 (3332)	5332 (6820)	<0.001
KIM-1 (pg/ml)	82.23 (94.05)	127.5 (157.76)	0.006
NGAL(ng/ml)	89.3 (48.3)	130.15 (92.07)	<0.001
Cys C (mg/L)	1.14 (0.31)	2.0 (0.95)	<0.001
NAG (nmol)	14.5 (4.9)	15.28 (8.51)	0.166
H-FABP (pg/ml)	1469.97 (1739.88)	2677.9 (4191.8)	<0.001
eGFR (mL/min/1.73 m^2^)	74.13 ± 1.07	43.23 ± 1.64	<0.001
miR-21	0.53 (0.44)	0.89(0.89)	<0.001

Data are expressed as Median (Quartile range) or Mean ± Standard deviation

*CRS*: type 2 Cardio-Renal syndrome; *miR-21*: MicroRNA-21; *CHF*: chronic heart failure; *eGFR*: estimated glomerular filtration rate; *KIM-1*: idney injury factor-1; *NGAL*: neutrophil gelatinase-related apolipoprotein; *Cys C*: cystatin C; *NT-proBNP*: amino-terminal pro-B-type natriuretic peptide; *NAG*: N-acetyl-κ-D-glucosaminidase; *H-FABP*: heart-typefatty acid–binding protein. *P* < 0.05 has statistically significant.

### Correlations of serum levels of miR-21 with cardiorenal syndrome biomarkers

**Figure 2:**
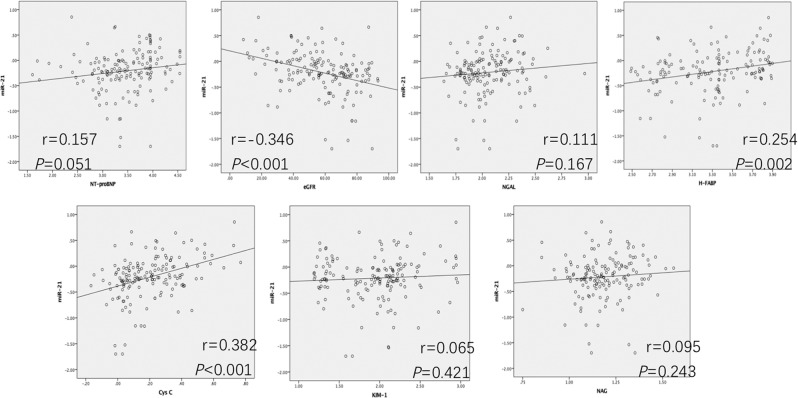
Correlations between miR-21 levels and other biomarkers. miR-21 levels by real-time PCR correlated significantly with Cys C, H-FABP and eGFR (except NAG, NT-proBNP, NGAL and KIM-1) in 157 elderly patients. The variates NT-proBNP, Cys C, NGAL, KIM-1, H-FABP,NAG and miR-21 were log-transformed in Pearson correlation.

Figure 2 shows that the levels of Cys C (r = 0.382, *P* < 0.001), H-FABP (r = 0.254, *P* = 0.002) and eGFR (r = −0.346, *P* < 0.001) correlated significantly with miR-21 expression. In contrast, correlations with miR-21 were not significant for NT-proBNP, NGAL, NAG and KIM-1.

### Multivariate analysis using logistic regression

Multivariate logistic regression was employed to estimate the independent relationshipbetween variables with significant differences (*p* < 0.1) in Tables 1 and 2 (hyperlipidaemia, HB, EF, NT-proBNP, NGAL, H-FABP, Cys C, KIM-1 and miR-21) and thepresence of CRS. Based on this analysis, serum miR-21 (OR: 17.246; 95% CI: 3.334–89.218; *P* = 0.001), increased serum Cys C (OR: 29.858; 95% CI: 9.588–92.983; *P* < 0.001), serum KIM-1 (OR: 4.041; 95% CI: 1.204–13.568; *P* = 0.024), hyperlipidaemia (OR: 8.415; 95% CI: 1.764–40.143; *P* = 0.008) and EF (OR: 0.940; 95% CI: 0.901–0.981; *P* = 0.004) were independent influencing factors for the occurrence of CRS (Table 3). Furthermore, multicollinearity was assessed among HB, EF, NT-proBNP, NGAL, H-FABP, KIM-1 and miR-21 by the variance inflation factor (VIF). None of the VIF values were greater than 10, and collinearity was not a problem. The Hosmer-Lemeshowtest showed significant goodness of calibration for the logistic model (*P* = 0.639).

**Table 3:** Multivariate logistic regression for identification of independent predictors of CRS.

Variables	B	S.E.	Wald	OR	95% CI	P-value
EF	−0.062	0.022	8.126	0.940	0.901–0.981	0.004
Hyperlipidemia	2.130	0.797	7.140	8.415	1.764–40.143	0.008
KIM-1^**#**^	1.397	0.618	5.108	4.041	1.204–13.568	0.024
miR-21^**#**^	2.848	0.839	11.532	17.246	3.334–89.218	0.001
Cys C						
<1.50 (mg/L)	Reference					
≥1.50 (mg/L)	3.396	0.580	34.342	29.858	9.588–92.983	<0.001

KIM-1^#^ = log(KIM-1); miR-21^#^ = log(miR-21).

OR, odds ratio; CI, confidence interval.

*miR-21*:MicroRNA-21; *Cys C*: cystatin C; *KIM-1*:kidney injury factor-1; *EF:* ejection fraction.

### The predictive value of miR-21 and combined biomarkers for cardiorenal syndrome

**Figure 3:**
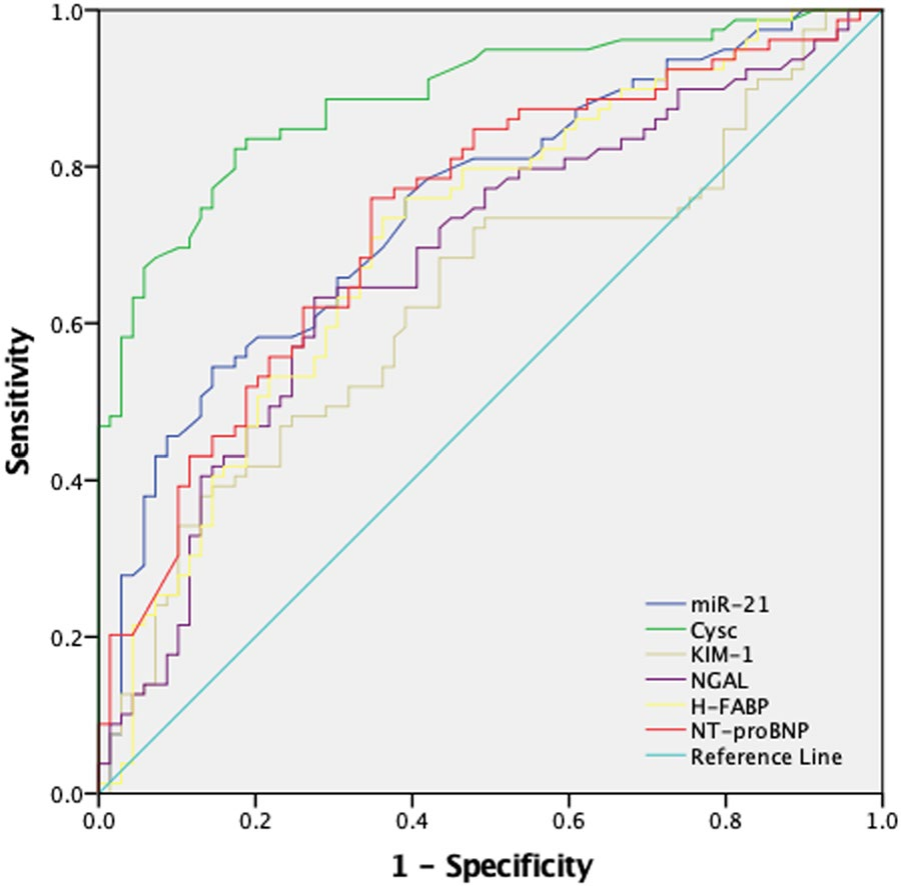
ROC analysis of individual biomarkers. KIM-1, NGAL, Cys C, NT-proBNP, H-FABP, NAG, and miR-21 were used to predict CRS in elderly CHF patients.

**Figure 4:**
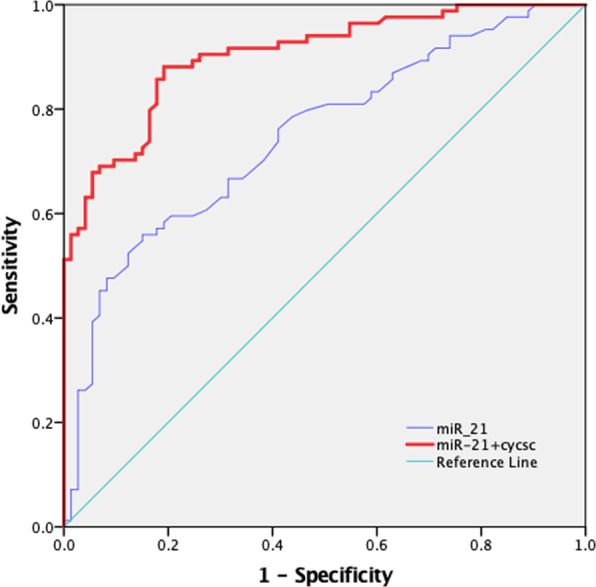
ROC analysis of the individual/combined biomarkers. Combined miR-21+CysC was used to predict CRS in elderly CHF patients compared to miR-21 alone.

The effect of miR-21 and combined biomarker groups on cardiorenal syndrome diagnosis is presented in Figs. 3 and 4. The results for the area under the receiver operating characteristic (ROC) curve, sensitivity, specificity, and Youden index value are presented in Table 4. For miR-21, we observed an area under the ROC curve of 0.749 (95% CI: 0.671, 0.827), a Youden index of 0.4088, a sensitivity of 55.95% and a specificity of 84.93%. Moreover, for the combined analysis, Cys C + miR-21 had good diagnostic value, with an area under the ROC curve of 0.902 (95% CI: 0.854, 0.950), which was better than that of miR-21 alone (*P* < 0.001).

**Table 4:** AUC for individual/combination biomarkers.

Biomarkers	The under area of ROC curve (95% CI)	sensitivity (%)	specificity (%)	Youden index
KIM-1	0.629 (0.539, 0.719)	39.29	87.67	0.27
NGAL	0.681 (0.595, 0.768)	63.10	73.97	0.37
Cys C	0.888 (0.836, 0.940)	83.33	82.19	0.66
NT-proBNP	0.736 (0.655, 0.816)	73.49	66.67	0.40
miR-21	0.749 (0.671, 0.827)	55.95	84.93	0.41
H-FABP	0.710 (0.626, 0.793)	72.50	64.29	0.37
Cys C + miR-21	0.902 (0.854, 0.950)	88.10	83.60	0.72
Cys C + KIM-1	0.891 (0.840, 0.942)	82.10	83.60	0.66
Cys C + NGAL	0.845 (0.784, 0.905)	67.90	93.20	0.61
CysC+ H-FABP	0.877 (0.823, 0.931)	66.30	95.70	0.62
Cys C + NT-proBNP	0.846 (0.786, 0.905)	84.30	72.20	0.57

Receiver operating characteristic (ROC) curve analysis was used to calculate the area under the curve (AUC) of individual and combined biomarkers for diagnosing CRS.

## Discussion

China is a gradually ageing society. CHF is the most common cardiovascular disease and is the most common cause of hospitalization in those older than 65 years. Furthermore, the incidence of CHF is 0.2–0.3% in the general population but increases greatly with age and is as much as 10-fold higher in those over 80 years of age^31^. CRS-2 is defined as renal impairment secondary to CHF, which is one of the leading causes of hospitalization, morbidity and mortality for elderly individuals with CHF and contributes greatly to the direct and indirect costs of CHF. Renal injury was typically an exclusion criterion in previous CHF clinical studies; thus, the reported evidence, diagnosis and management of organ pathology were not sufficient^1^. Accordingly, the detection of biomarkers, early diagnosis and timely treatment are particularly important for CRS-2 patients.

For patients with CHF, we analysed dynamic changes in serum NT-proBNP expression levels to evaluate cardiac function and assessed the expression levels of serum creatinine (Scr) and blood urea nitrogen (BUN) to estimate kidney damage. However, the heart-kidney interaction in CRS-2 patients causes a series of changes in neurohormones and inflammatory factors, and the internal environment changes significantly. Thus, assessment of disease status cannot be limited to the evaluation of a single organ’s function. Moreover, when Scr and BUN abnormalities occur in patients, serious renal function damage has already occurred, suggesting that Scr and BUN are relatively late biomarkers. Some new biomarkers, such as NGAL, Cys C, KIM-1, H-FABP and NAG, have been reported to have diagnostic and prognostic value in CRS-2 patients^7^. For example, NGAL is considered an excellent biomarker for the early prediction of CKD^32^, and Bolignano *et al*.^33^ suggested that NGAL could serve as a biomarker of atherosclerosis or CVD as well as CKD progression. Jungbauer *et al*.^34^ followed CHF patients for 5 years and found a strong association of the tubular biomarkers KIM-1 and NAG with CKD progression in CHF, which may suggest their suitability as cardiorenal biomarkers. Cys C also appears to be of value in cardiovascular risk stratification of elderly patients with metabolic syndrome^35^, and H-FABP has diagnostic value in ACS^36^. Moreover, Oezkur *et al*. identified a correlation between preoperative serum H-FABP and the postoperative incidence of renal impairment in ACS patients^37^. In summary, NGAL, Cys C, KIM-1, H-FABP and NAG are considered to be effective in the early detection of renal impairment and may also reflect cardiac function in CRS-2 patients, contributing to the early diagnosis, prevention and treatment of CRS-2^7,38^.

Many studies to date suggest that circulating miRNAs can be applied as biomarkers for disease diagnosis/prognosis, as well as therapeutic targets for certain diseases^12,13,39^. miRNAs are endogenous noncoding small RNAs with regulatory functions that recognize target RNAs by complementary base-pairing and guide silencing complexes to degrade the target mRNA or repress its translation. miRNAs also play important roles in the occurrence and development of various diseases and the ageing of organs, and circulating miRNAs stably reflect gene regulation through the internal environment of an individual. Thus, by evaluating circulating miRNAs, the overall assessment of diseases becomes more effective and accurate. Among numerous miRNAs, studies have shown that miRNA-21 plays a regulatory role in both heart and kidney function. Located on chromosome 17, miRNA-21 is a member of the mitochondrial family subtype, with its own specific promoter^40^. miR-21 participates in the pathological and physiological processes of many diseases, including cardiovascular diseases, in which it prevents post-infarction cardiac insufficiency through targeted regulation^16^ and participates in the process of atherosclerosis by mediating signalling^41^. One study reported that high expression of miRNA-21 can promote fibroblast activation and accelerate the progression of renal fibrosis. In addition, knockdown of miR-21 expression in animal models through a gene modification technique effectively inhibited the progression of renal fibrosis caused by renal injury^42^. A high level of miRNA-21 downregulates the expression of its targets and accelerates the progression of cardiac fibrosis; therefore, it is an important signalling molecule for cardiac fibrosis^17^. This evidence supports the possibility of using miR-21 for the early diagnosis and assessment of CRS-2.

In our present study, we found significant differences in the abovementioned biomarkers between the CRS and CHF groups. In addition, Cys C, H-FABP and eGFR correlated significantly with miR-21 expression. In multivariate regression analysis, serum miR-21, increased serum Cys C, serum KIM-1, hyperlipidaemia and EF were independent influencing factors for CRS (*P* < 0.05), confirming previous research results and our experimental hypothesis. The highest AUC value, 0.888, was observed for Cys C, indicating that Cys C has particular advantages for prediction and early diagnosis. Cys C is a cysteine protease inhibitor, and its half-life is only 1.5 h (for comparison, Scr’s half-life is 4 h)^43^. The level of Cys C only depends on the glomerular filtration ability, and experiments have shown that the biological change in Cys C is only 7% of that in Scr^6^. Therefore, compared with other biomarkers, Cys C better reflects changes in renal function^44^. Our results also showed that miR-21 expression was significantly increased in the CRS group, with elevated miR-21 being an independent risk factor for CRS. Furthermore, miR-21 plays a role in the diagnosis of CRS. Among the tested biomarkers, miR-21 was second only to Cys C: the AUC for CRS-2 was 0.749, and its specificity was 84.93%, which was better than that of Cys C (82.19%), although this difference was not statistically significant (*P* > 0.05). Indeed, the miR-21 AUC for CRS-2 was higher than that of most biomarkers (*P* < 0.001), except Cys C. These results indicate that miR-21 has an important function in the occurrence and development of CRS disease. Additionally, gene regulation related to miR-21 may be studied to find an effective basis for gene regulation therapy in CRS patients and to explore new treatment schemes, which will slow CRS disease development and contribute to effective intervention. In general, regulation of miR-21 may inhibit or delay the progression of cardiac and renal fibrosis and improve the prognosis of CRS-2.

Regarding our combined diagnostic analysis, although the diagnostic value of miR-21 (AUC 0.749; 95% CI: 0.671–0.827) was not higher than that of Cys C (AUC 0.888; 95% CI: 0.836–0.940), the combined AUC of these biomarkers for CRS-2 was 0.902 (95% CI: 0.854–0.950), with the highest sensitivity in the model, at 88.1%, representing a great improvement in the diagnostic value for CRS-2 compared with that of miR-21 alone (*P* < 0.001). This suggests that in addition to eGFR, dynamic changes in Cys C and miR-21 may facilitate the diagnosis/evaluation of CRS diseases and the determination of the disease status of these patients.

## Conclusion

Serum circulating miR-21 is increased in patients with CRS-2. Circulating miR-21 has medium diagnostic value; therefore, it may become a factor in the clinical pathophysiological activation and diagnosis of CRS-2. The combined assessment of miR-21 and Cys C has good clinical value for CRS-2, with a potential role in the clinic for better monitoring the status of CRS patients.

## Methods

### Ethics statement

All experimental procedures were approved by the Ethics Committee of Hebei General Hospital [clinical ethics approval number 2017203]. The study was conducted in accordance with the Declaration of Helsinki and with Good Clinical Practice guidelines, as defined by the International Conference on Harmonisation. All patients provided written informed consent before enrolment. A data and safety monitoring committee was established to provide oversight of safety and efficacy considerations. The study was registered on ResMan [approval number ChiCTR1800016858].

### Study participants

We applied the following inclusion criteria: (i) age over 65 years; (ii) NYHA II-IV; (iv) occurrence of cardiac insufficiency earlier than renal insufficiency in CRS patients. Exclusion criteria: patients with autoimmune diseases, cancer, liver diseases, haematological diseases, recent surgery or trauma, acute and/or chronic inflammatory state, and CHF induced by primary kidney disease or CKD (i.e., CRS-4). Data for a total of 157 patients diagnosed with CHF at Hebei General Hospital between November 2017 and March 2019 were collected, including 74 males and 83 females between 65 and 97 years old. Fresh blood samples were analysed using standard hospital assays to measure serum levels of creatinine, NGAL, Cys C, NT-proBNP. The estimated glomerular filtration rate (eGFR) was calculated by the CKD-EPI Creatinine 2009 Equation, and the 157 patients were divided into a CRS group (eGFR < 60 ml/min/1.73 m^2^) and a CHF group (eGFR ≥ 60 ml/min/1.73 m^2^).

### Sample preparation

We collected 3–4 ml peripheral blood from each patient into a procoagulation tube (HEBEI XINLE SCI&TECH CO., Ltd, Hebei, China), and the sample was centrifuged at 2000 rpm for 10 min. The supernatant was collected into a clean EP tube and further centrifuged at 13,000 rpm for 10 min to remove intact chromatin from ruptured blood cells. The supernatant was collected into another clean EP tube and stored at −80 °C.

### Quantitative real-time polymerase chain reaction

MiRNAs were extracted from the prepared blood using miRNeasy Serum/Plasma Advanced Kit (Qiagen, Germany) strictly following the manufacturer’s instructions. Quantitative RT-PCR was performed as described in the manual (Qiagen, Germany). The primers for miRNA used in this study were purchased from Qiagen. In our pilot experiment, some of our samples (approximate from 18 CHF/CRS patients) were tested both synthetic spike-in controls (unisp2/unisp4/unisp5 unisp6/cel-mir-39 and unisp3) (Qiagen, Germany) and miR-103a at the same time. The results showed (As Supplementary Figure) that ΔCT between miR-103a and cel-mir-39 (commonly used as synthetic exogenous reference RNA) was relatively stable, suggesting that serum miR-103a in our CHF/CRS patients was relatively stable, might be used for normalization in our experiment as an endogenous reference miR. Thus, the level of miR-21 was normalized to that of miR-103a, and melting curves were employed to analyse sample specificity. The specimen with the lowest Ct for the miR-21 gene was used as a control. The results for each serum specimen were calculated with the 2^−ΔΔCt^ method. That is, every sample was characterized by the difference in the threshold cycle (CT) between miR-21 and miR-103a: ΔCt = Ct_miR-21_ − Ct_miR-103a_. Relative expression of the miRNA gene was calculated with 2^−ΔΔCt^.

### KIM-1 H-FABP ELISA and NAG activity assay

The serum levels of KIM-1 (R&D, USA) and H-FABP (Abcam, UK) and NAG activities (Abcam, UK) were measured by commercially available ELISA and activity assay kits following the manufacturer’s recommendations. Briefly, for the KIM-1 and H-FABP ELISAs, 50 μl of serum sample was added to plates precoated with a monoclonal antibody and incubated for 2 h at room temperature. After washing three times with wash buffer, 200 μl human serum KIM-1/H-FABP conjugate antibody was added to each well and incubated for another 2 h. After washing 3 times with buffer, the plates were incubated with 200 µl substrate solution for 30 min, and 50 µl stopping buffer was added to terminate the reaction. Serum KIM-1/H-FABP concentrations were assessed by detecting absorbance at 450 nm using an ELISA instrument (Thermo FC, US). For the serum NAG activity assay, 70 μl of serum sample was added to the plates, after which 55 μl of NAG substrate was added and incubated for 30 minutes; the reaction was terminated with 25 µl of stopping buffer, and the plate was incubated for 10 minutes in the dark. The serum NAG activity was calculated based on absorbance detection at OD 400 nm.

### Statistical analysis

Data were analysed using SPSS version 22.0 (SPSS Inc.; Chicago, IL, U.S.A.). The data from the two groups did not have a normal distribution. Categorical data are expressed as ratios or percentages, and the chi-square test was conducted to compare groups. Continuous measurement data are displayed as the median (quartile range). Differences between the two groups were analysed with the Mann-Whitney U test. Associations between biomarker expression and miR-21 were analysed by Pearson correlation. To identify biomarkers with potential significant contributions to CRS, binary logistic regression and multiple forward stepwise logistic regression were constructed with the variables with significant differences (p < 0.1) in Tables 1 and 2. The biomarkers were log-transformed in Pearson correlation and logistic regression analyses. Moreover, Cys C was divided into two groups: Cys C < 1.50 mg/L and Cys C ≥ 1.50 mg/L in the logistic model. Multicollinearity was examined using the VIF among the included continuous variables in the logistic model. If none of the VIF values are greater than 10, collinearity is not a problem. Calibration of the logistic model was assessed using the Hosmer-Lemeshow test, whereby we considered a value of *P* < 0.05 to indicate that the model had poor calibration. ROC curves were constructed with the individual/combination biomarkers, and the AUC, sensitivity, specificity, and Youden index were applied to estimate the predictive value of the biomarkers. The improvement between two ROC curves was calculated by Delong’s test, and statistical significance was defined as two-tailed *p* < 0.05.

### Consent for publication

Each patient signed informed consent before inclusion in the trial, including acceptance for data publication.

### Supplementary information

ΔCT between miR-103a and cel-mir-39 in the pilot experiment.

## Data Availability

The datasets used and/or analyzed during the current study are available from the corresponding author upon reasonable request.
