# Supplementary material for: Circulating miRNA-21 as a diagnostic biomarker in elderly patients with type 2 cardiorenal syndrome
Source: Sci Rep. 2020 Mar 17;10:4894. doi: 10.1038/s41598-020-61836-z (PMC7078306; doi:10.1038/s41598-020-61836-z)
Supplement: Supplementary file 1 — ΔCT between miR-103a and cel-mir-39 in the pilot experiment. [file 41598_2020_61836_MOESM1_ESM.docx]

***Supplementary Information***

**Circulating miRNA-21 as a diagnostic biomarker**

**in elderly patients with type 2 cardiorenal syndrome**

**Author:**

Yan Wang^1*^, Yi Liang^1,2#^, WenJun Zhao^2,4#^, GuangPing Fu^3^, QingQuan Li^1^, XuChen Min^1^, YiFang Guo^1*^

*Correspondence: wy890125fgp@163.com; guoyifang@hotmail.com.

^#^*Contributed equally to this work*

**Affiliations:**

^1^Department of Geriatric Cardiology, Hebei General Hospital, Shijiazhuang, Hebei, China

^2^Hebei Medical University, major in Cardiovascular Medicine, Shijiazhuang, Hebei, China

^3^Hebei Key Laboratory of Forensic Medicine, Department of Forensic Medical, Hebei Medical University, Shijiazhuang, Hebei, China

^4^Department of International Medical, the First Hospital of Shijiazhuang, Shijiazhuang, Hebei, China

**Supplementary Figure**


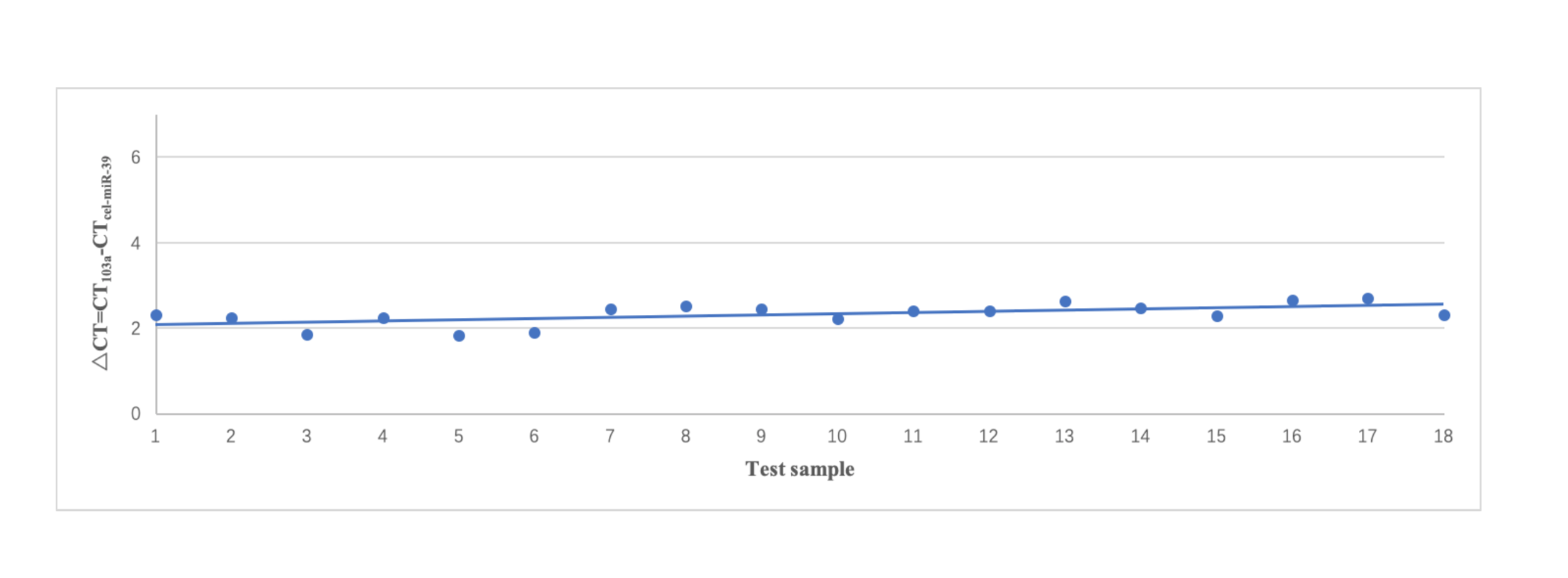


***Fig. S*** *ΔCT between miR-103a and cel-mir-39 in the pilot experiment*
